# Wernicke's encephalopathy after acute pancreatitis with upper gastrointestinal obstruction: A case report and literature review

**DOI:** 10.3389/fneur.2023.1108434

**Published:** 2023-02-23

**Authors:** Zongding Wang, Lei Zhang, Xingzhen Deng, Zili Peng, Shaoyong Liang

**Affiliations:** ^1^Department of Hepatobiliary Surgery, Fengjie County People's Hospital of Chongqing, Chongqing, China; ^2^Department of Hepatobiliary Surgery, Fengjie Hospital, The Second Affiliated Hospital of Chongqing Medical University, Chongqing, China

**Keywords:** acute pancreatitis, vitamin B1, Wernicke's encephalopathy, case report, gastrointestinal obstruction

## Abstract

A 42-year-old female was admitted with upper abdominal pain. Imaging studies and laboratory tests were performed to consider acute lipogenic pancreatitis. After symptomatic treatment, her abdominal pain was significantly relieved. However, the patient was accompanied by upper gastrointestinal obstruction, which was gradually relieved after long-term fasting, gastrointestinal decompression, and fluid rehydration. The patient developed dizziness and ataxia, which worsened. Cranial magnetic resonance imaging (MRI) indicated patchy abnormal signal shadows in the bilateral thalami and dorsal brainstem and suggested metabolic encephalopathy. Wernicke's encephalopathy (WE) was the initial diagnosis of suspicion, adequate vitamin B1 was immediately replenished until the complete resolution of symptoms, and the patient made a rapid and dramatic recovery.

## Background

Acute pancreatitis is a serious disease with high mortality and relapse rates (1). The etiology of pancreatitis includes gallstones and alcohol, followed by hyperlipidemia. Acute pancreatitis includes acute edematous pancreatitis and acute necrotizing pancreatitis. The latter is prone to cause a variety of complications, such as hemorrhage, necrosis, infection, systemic inflammatory response syndrome (SIRS), acute respiratory distress syndrome (ARDS), and multiple organ dysfunction syndrome (MODS) (2). Additional complications that have been reported include pancreatic encephalopathy, intestinal fistulae, and upper gastrointestinal tract obstruction, which makes the treatment of acute pancreatitis difficult (3). Persistent upper gastrointestinal obstruction due to acute pancreatitis has rarely been described.

Obstruction of the entrapped bowel is a surgical emergency that requires timely surgery to relieve the obstruction. The most common clinical manifestations of upper gastrointestinal obstruction are abdominal pain, poor appetite, nausea and vomiting, and it predisposes patients to ischemia and perforation with a high mortality if left untreated (4). Such obstruction, when persistent, results in a huge loss of body fluid and chronic undernutrition that may induce changes in physiological function (5).

Vitamin B1 is an important cofactor in glucose metabolism and plays a crucial role in enzymatic systems that require its active form (6). Wernicke encephalopathy is a metabolic disease mainly associated with single vitamin B1 deficiency that leads to lactic acid accumulation and permanent brain injury causing neuropsychiatric symptoms. Without prompt management, it has the potential to cause serious, life-threatening complications and even mortality (7). Therefore, the primary prerequisite for enhancing the diagnosis of Wernicke encephalopathy is that an early and accurate diagnosis is accepted to be a highly significant issue. Herein, we report a case of Wernicke encephalopathy after acute pancreatitis in a patient with upper gastrointestinal obstruction.

## Case presentation

**Figure 1:**
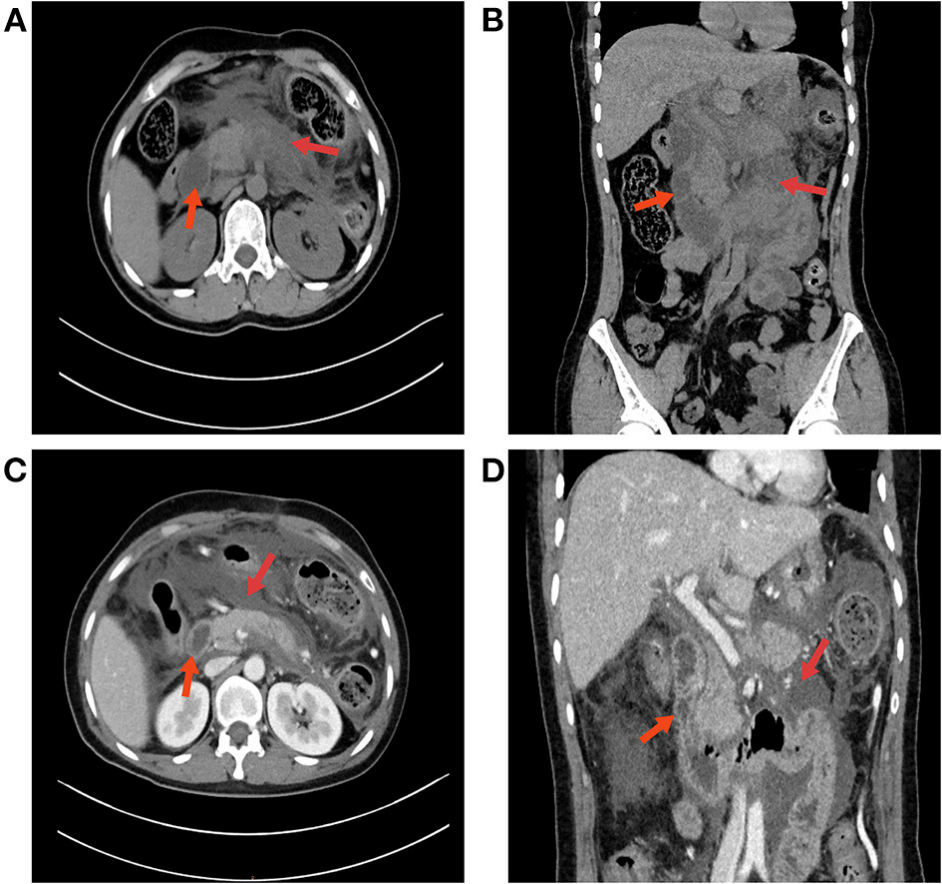
An abdominal CT scan confirmed the diagnosis of acute pancreatitis. **(A)** Abdominal CT revealed fluid collection around the pancreas; red arrows indicate an undilated duodenum and a large amount of exudate. **(B)** CT coronal imaging. **(C)** Contrast-enhanced abdominal CT. The arrow on the left indicates the duodenum, and the arrow on the right indicates a necrotic lesion of the pancreas with a large amount of local exudate. **(D)** No obstruction was found in the duodenum on abdominal CT.

A 42-year-old female presented with a 2-day history of persistent abdominal pain in the upper abdomen, accompanied by nausea and decreased appetite. The patient did not receive any medications prior to admission. A physical examination revealed tenderness of the left upper quadrant of the abdomen. She had a history of acute necrotizing pancreatitis due to gallstones and had undergone laparoscopic cholecystectomy 5 years prior. Subsequent follow-up did not reveal biliary stones. She was never a smoker, did not consume alcohol, and denied drug abuse. She had no history of hereditary diseases or relevant family history. Her BMI was 21.23. Abdominal computed tomography (CT) revealed fluid collection around the pancreas and peripancreatic inflammation with diffuse pancreatic edema (Figure 1). Relevant laboratory findings were as follows: hemoglobin was 107.0 g/dl (reference range: 115.0–150.0 g/dl), white blood cell count was 10.9 × 10^9^/L (reference range: 3.50–9.50 × 10^9^/L), platelet count was 215.0 × 10^9^/L (reference range: 125.0–350.0 × 10^9^/L), the international normalized ratio was 0.9 (reference range: 0.8–1.5), glucose was 9.03 mmol/L (reference range: 3.90–6.10 mmol/L), blood urea nitrogen was 4.50 mmol/L (reference range: 2.60–7.50 mmol/L), blood amylase was 1,480 U/L (reference range: 35–135 U/L), lipase was 500 U/L (reference range: 0–60 U/L), and plasma triglyceride levels were significantly increased (12.38 mmol/L, reference range: 0–1.70 mmol/L). The initial laboratory tests showed that serum liver function and tumor biomarkers were normal. A diagnosis of acute hyperlipidemic pancreatitis was made, and the patient was admitted. The patient was treated with liquid resuscitation, intravenous injection of trypsin inhibitors, and maintenance of water and electrolyte balance.

**Figure 2:**
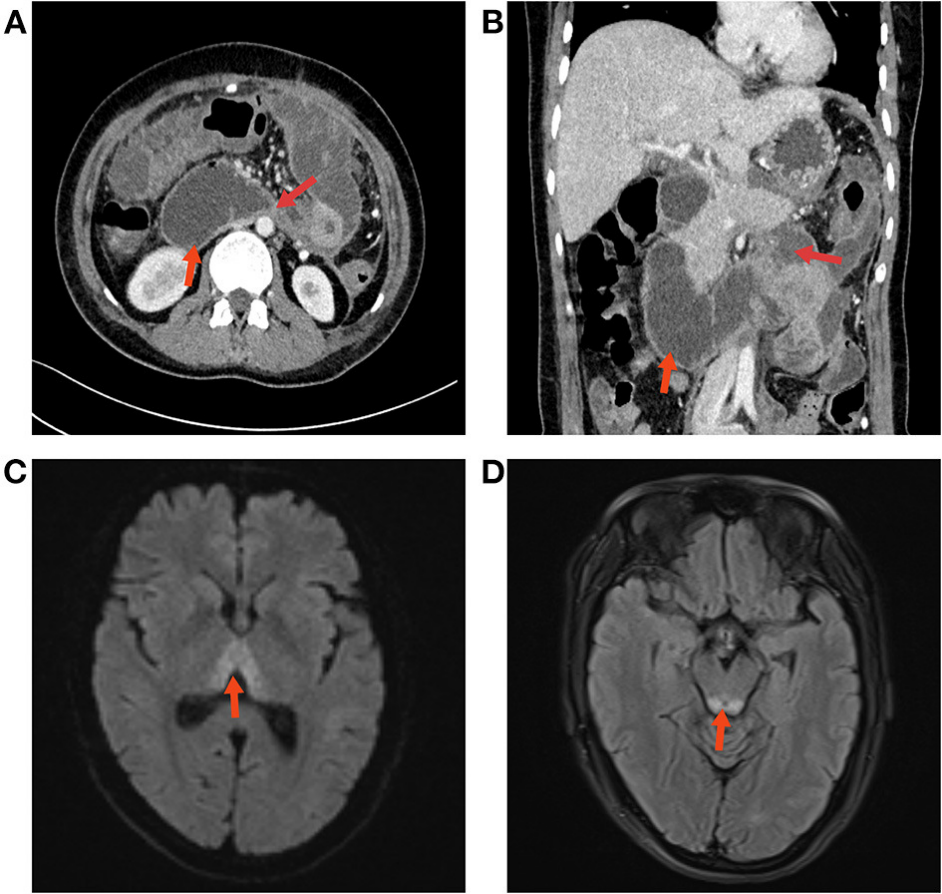
**(A, B)** Abdominal CT re-examination showed duodenal obstruction and proximal dilatation. **(C)** The results of the cranial MRI suggested patchy abnormal signal shadows in the bilateral medial thalamic region. **(D)** Edema was evident in the dorsal area of the brain stem around the aqueduct.

One week after admission, the patient's abdominal pain was gradually relieved and she complained of increasing abdominal distention. Moreover, the patient was unable to eat and was simultaneously suffering from nausea and bilious vomiting. She developed moderate hyponatremia (131 mmol/L, reference range: 137.0–147.0 mmol/L) with hypochloremia (91.5 mmol/L, reference range: 99.0–110.0 mmol/L). An abdominal CT re-examination showed complete obstruction of the horizontal duodenum, dilatation of the proximal small intestine and stomach, and increased peripancreatic edema (Figures 2A, B). She received conservative treatments with acid suppression, fasting, gastrointestinal decompression and nutritional support. The patient was subjected to fasting and gastrointestinal decompression.

**Figure 3:**
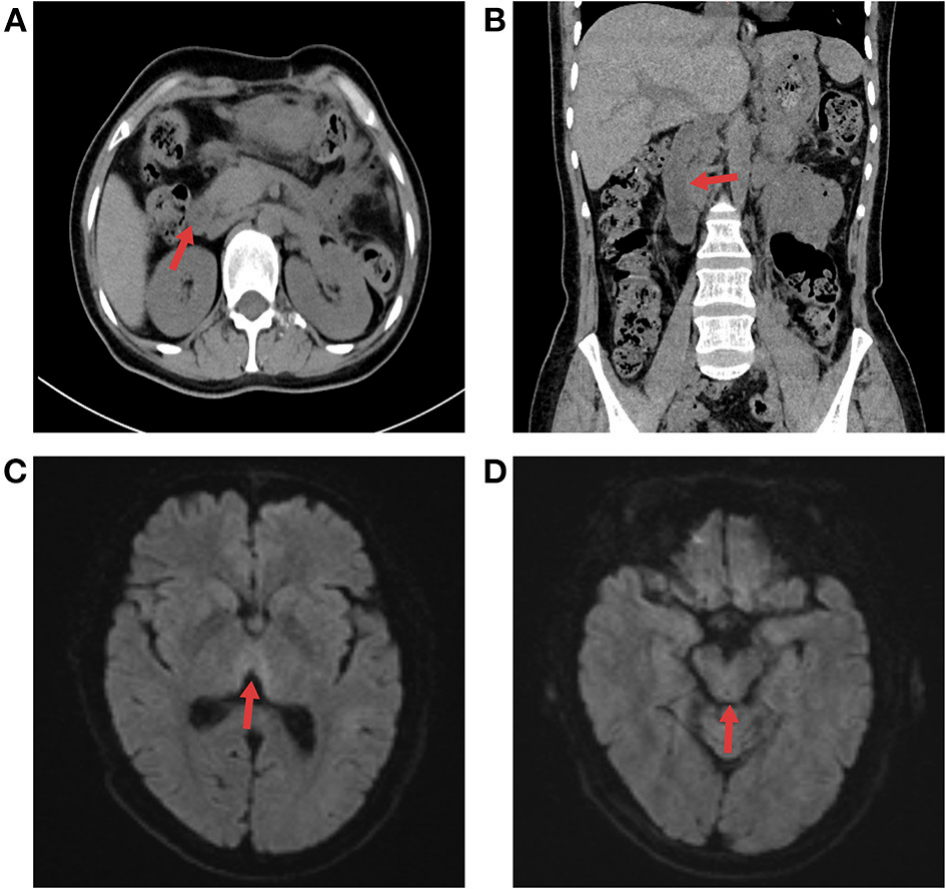
The follow-up radiographic results. **(A, B)** Abdominal CT showed that pancreatic edema subsided, peripancreatic exudate was obviously absorbed, and the duodenum was not dilated or obstructed. **(C, D)** Cerebral MRI results at 3 months after discharge indicated that edema had disappeared in the medial thalamus and dorsal brain stem.

During this period, she received 10% hypertonic saline supplementation *via* a nasogastric tube (30 ml twice daily). After 1 week of symptomatic and supportive treatment, the patient improved. Her abdominal distension eased, and her gastrointestinal function and diet gradually returned. Then, the patient complained of dizziness, acute gait unsteadiness and clumsy walking, which slowly progressively worsened, without nystagmus, headache or vomiting. Physical examination showed that she was conscious, her neck was supple, her meningeal signs were negative, and she had normal limb muscle strength. Cranial MRI indicated patchy abnormal signal shadows in the bilateral thalami and dorsal brainstem and suggested metabolic encephalopathy (Figures 2C, D). Liver and renal function tests, urinalysis, electrolytes, coagulation, blood sugar, white blood cell counts, procalcitonin (PCT), and microelement (Mg, Cu, Fe, Zn, Mn, Sr, and Ca) studies were within normal limits. Previous fluid supplementation revealed no vitamin B1 supplementation. The level of vitamin B1 was also examined (0.8 ng/ml, reference range: 1.0–10.0 ng/ml). An initial diagnosis of WE was made. Therefore, vitamin B1 (700 mg/day) was immediately supplemented intravenously for 5 days, and the total glucose infusion was reduced. Shortly afterward, her condition markedly improved; she could walk normally, she resumed a normal diet, and she was asymptomatic. Furthermore, no abdominal distension or diarrhea was observed. The vitamin B1 administration was later changed to oral supplementation (100 mg/day). She was regularly followed up for 3 months after discharge, and no abnormalities were detected during the follow-up (Figure 3).

## Discussion and conclusion

WE was first described in chronic alcoholic liver disease (8) but has occasionally been reported in patients with chronic malnutrition due to a variety of causes, including weight-loss gastrectomy and hyperemesis gravidarum (9). Chronic malabsorption of nutrients resulting in WE due to acute pancreatitis with upper gastrointestinal obstruction has rarely been reported. Additionally, inadequate vitamin B1 supplementation is correlated with contributing to this result. Vitamin B1 plays an essential role in intracellular glucose metabolism, and its deficiency leads to cell edema, especially in the central nervous system (10). However, neither the electroencephalograph (EEG) nor neurological examination results revealed any pathological findings. Fortunately, the symmetry variability of MRI is excellent for the diagnosis of metabolic encephalopathy (7). The patient presented with typical neurological symptoms, including dizziness, lethargy and ataxia. The estimation of vitamin B1 in blood further confirmed the previous preliminary diagnosis. Furthermore, it is worth mentioning that vitamin B1 supplementation by enteral or parenteral routes might interfere with the assay results.

If the required vitamin B1 is not obtained, the degree of brain congestion and edema will be aggravated in WE, even necrosis (11). Obviously, the basal ganglia and thalamic region seem to be particularly vulnerable to oxygen deprivation according to the reported literature (12, 13). Accordingly, the corresponding clinical symptoms of WE appeared in this patient. In addition, some symptoms of WE are atypical and are easily missed or misdiagnosed, which brings diagnostic challenges to clinicians. Furthermore, severe clinical signs might develop, including respiratory and circulatory failure (9, 14). As a result, delayed diagnosis in patients with WE is associated with high mortality. Hence, it is important to make a timely diagnosis of WE. Vitamin B1 supplementation should not wait or be based on imaging studies or laboratory results. In differential diagnosis, it is necessary to distinguish WE from pancreatic encephalopathy, which is mainly related to toxic substances released by pancreatitis and pancreatic enzymes (15), and it needs to be differentiated from viral encephalitis (16), hepatolenticular degeneration (17), and subacute necrotizing encephalitis (18) as well.

Regarding the analysis of etiologies, there are many factors that contribute to vitamin B1 deficiency, including chronic alcoholism, hyperemesis gravidarum, food-induced malnutrition, bariatric gastrectomy, and prolonged fasting (19). Meanwhile, acute pancreatitis may be accompanied by local complications in the later stage, such as pseudocysts, infectious necrotizing pancreatitis, and intestinal fistula, and a few patients will experience intestinal obstruction (3, 20). In addition, cases of intestinal obstruction due to acute pancreatitis have rarely been reported. This is a very unusual case of upper gastrointestinal obstruction followed by WE caused by vitamin B1 deficiency. Hence, depending on a history of long-term fasting and lack of timely nutritional supplementation of vitamin B1, we initially suspected the disease to be WE, which was verified by MRI and laboratory test data.

Pancreatic encephalopathy and WE are rare complications of acute pancreatitis. Without timely and accurate treatment, patients have little chance of survival (21). The distinction between pancreatic encephalopathy and WE should not be neglected. Pancreatic encephalopathy mainly occurs in the early stage of severe acute pancreatitis. The pathogenesis of pancreatic encephalopathy is complex. Current findings suggest that pancreatic encephalopathy is mainly associated with the release of phospholipase A2 and lipase from the pancreas into the blood and through the blood-brain barrier, leading to brain tissue damage, including edema, hemorrhage, and demyelination (21–24). In addition, some studies have also showed that the release of large amounts of inflammatory mediators in severe acute pancreatitis increases permeability and destroys the blood-brain barrier (25, 26). It has also been reported to be associated with hemodynamic instability and severe electrolyte disturbances (27). Besides, some case reports have also found that acute pancreatitis is also prone to WE, which is related to vitamin B deficiency (28–30). It mainly occurs in the later stages of pancreatitis. Both of these complications are serious threats to the life safety of patients, because the clinical manifestations are not specific, complications progress quickly, which are irreversible in the advanced stage. The specific mechanism of these complications in acute pancreatitis is still unclear and needs data support.

In terms of treatment, etiological treatment is the most important treatment approach for this case. The key points in the early stages of acute pancreatitis are rehydration, hypolipemic drug therapy, low molecular weight heparin and insulin (31, 32). In the current diagnosis and treatment guidelines for acute pancreatitis, timely supplementation of vitamin B1 is not clearly mentioned. The supportive treatment of acute pancreatitis is very important, and early eating is advocated, which is conducive to the early recovery of gastrointestinal function (33, 34). However, the patient's abdominal pain improved after symptomatic treatment but then developed abdominal distension due to upper gastrointestinal obstruction. After conservative treatment, the obstruction was relieved, and the diet gradually resumed. However, prolonged fasting contributed to vitamin B1 deficiency, which eventually led to WE. Fortunately, after supplementing vitamin B1 in time, the symptoms were relieved, without leaving any sequelae. To prevent recurrence of acute pancreatitis, controlling the triglyceride level and outpatient follow-up after discharge are of critical importance, otherwise, it can easily relapse.

In conclusion, acute pancreatitis combined with upper gastrointestinal obstruction leads to WE caused by vitamin B1 deficiency, which is prone to misdiagnosis, missed diagnosis and delayed treatment. Patients with chronic fasting or malnutrition should consider vitamin B1 supplementation.

## Data availability statement

The original contributions presented in the study are included in the article/supplementary material, further inquiries can be directed to the corresponding author.

## Ethics statement

Ethical review and approval was not required for the study on human participants in accordance with the local legislation and institutional requirements. The patients/participants provided their written informed consent to participate in this study. Written informed consent was obtained for the publication of this case report.

## Author contributions

LZ and XD treated the patient. SL consulted the patient. ZP interpreted the CT and MRI findings. ZW wrote the first manuscript. All authors were involved in the analysis, interpretation of findings, proofed the manuscript, contributed to important intellectual content and writing, and approved the final manuscript.
